# Investigating Endogenous Opioids Unravels the Mechanisms Behind Opioid-Induced Constipation, a Mathematical Modeling Approach

**DOI:** 10.3390/ijms26136207

**Published:** 2025-06-27

**Authors:** Celvic Coomber, Surahit Chewle, Christopher Secker, Konstantin Fackeldey, Marcus Weber, Stefanie Winkelmann, Christof Schütte, Vikram Sunkara

**Affiliations:** 1 Institute of Mathematics, Technische Universität Berlin, 10623 Berlin, Germany; fackeldey@zib.de; 2 Zuse Institute Berlin, 14195 Berlin, Germanyweber@zib.de (M.W.);; 3 Department of Mathematics and Computer Science, Freie Universität Berlin, 14195 Berlin, Germany; 4 Deutsches Rheuma-Forschungszentrum, 10117 Berlin, Germany

**Keywords:** mathematical modeling, opioid-induced constipation (OIC), enteric nervous system (ENS), Endomorphin-2

## Abstract

Endogenous opioids, such as Endomorphin-2, are not typically associated with severe constipation, unlike pharmaceutical opioids, which induce opioid-induced constipation (OIC) by activating *μ*-opioid receptors in the gastrointestinal tract. In this study, we present a mathematical model, which integrates the serotonergic and opioid pathways, simulating the interaction between serotonin and opioid signaling within the enteric nervous system (ENS). The model explores the mechanisms underlying OIC, with a focus on the change in adenylyl cyclase (AC) activity, cAMP accumulation, and the distinct functionalities of Endomorphin-2 compared to commonly used pharmaceutical opioids. We study the effects of Morphine, Fentanyl, and Methadone and contrast them with Endomorphin-2. Our findings reveal that opioids do not perturb the signaling of serotonin, but only the activity of AC, suggesting that serotonin levels have no influence on improving opioid-induced constipation. Furthermore, this study reveals that the primary difference between endogenous and pharmaceutical opioids is their degradation rates. This finding shows that modulating opioid degradation rates significantly improves cAMP recovery. In conclusion, our insights steer towards exploring opioid degrading enzymes, localized to the gut, as a strategy for mitigating OIC.

## 1. Introduction

G protein-coupled receptors (GPCRs) represent the largest class of membrane receptors in the body, found across nearly every tissue [1,2]. These receptors are coupled to G proteins and play a vital role in mediating cellular responses to a wide variety of stimuli. Given their abundance and critical function, GPCRs are the target of approximately 40% of all drugs on the market.

A prominent example of a GPCR is the *μ*-opioid receptor (*MOR*), which is involved in pain modulation. The class of *μ*-opioid receptors binds to opioids, a group of potent pain-relieving medications, used to treat both chronic and acute pain. However, despite their efficacy, opioids are associated with numerous side effects, such as dependence, respiratory depression, nausea, or constipation, which is the most common among them [3,4,5,6]. Opioid-induced constipation (OIC) affects approximately 40% to 95% of patients treated with opioids [7,8]. This occurs due to the activation of *μ*-opioid receptors in the enteric nervous system (ENS) [8,9]. The ENS, akin to the central nervous system, comprises a network of interacting neurons, including motor neurons, secretomotor neurons, and vasodilator neurons, which regulate bowel contractions, secretion levels, and defecation frequencies [10,11].

A key player in the upkeep of a healthy gut is the neurotransmitter serotonin (*5HT*) [12,13,14]. The neurotransmitter *5HT* is synthesized in enterochromaffin (EC) cells of the intestinal epithelium from the amino acid tryptophan and is released in response to mechanical or chemical stimuli [12]. Upon release, the neurotransmitter *5HT* binds to various serotonergic receptors, including the 5HT-1, 5HT3, and 5HT-4 receptors (*5HTR4*) which are located on various neurons of the ENS [15]. The group of 5HT-4 receptors also belongs to the class of GPCRs such as the *μ*-opioid receptor and is known to play a crucial role in regulating gut motility [14,15]. For this reason, this group of receptors will be a primary focus in this paper.

Both *μ*-opioid receptors and 5HT-4 receptors act on the common effector adenylyl cyclase (*AC*), an enzyme responsible for synthesizing cyclic AMP (*cAMP*). As a critical secondary messenger, *cAMP* plays a vital role in various downstream processes, including the regulation of gut motility [16].

Activation of 5HT-4 receptors enhances the activity of the enzyme *AC* via G_*s*_ proteins, resulting in increased *cAMP* production [17]. Contrarily, *μ*-opioid receptors inhibit the activity of the enzyme *AC* through G_*i*_ proteins, leading to a reduction in *cAMP* levels [18,19]. Thus, treatments with opioids result in perturbed enzymatic activity of *AC* and reduced *cAMP* levels. In the context of pain management, this reduction in *cAMP* levels is one of several key mechanisms of opioid analgesia, alongside the closing of $K^{+}$ and $Ca^{2+}$ channels. While crucial for analgesia, decreased *cAMP* levels among other factors in non-target tissue, such as the gastrointestinal tract, can result in unwanted side effects, like constipation. In a healthy state, the gut contracts in a rhythmic motion, which is maintained by the neurotransmitter *5HT* being released in bursts and activating 5HT-4 receptors [15]. The activated 5HT-4 receptors act positively on the enzyme *AC* via their G_*s*_ proteins, increasing the *cAMP* production and leading to subsequent muscle contraction. On the other hand, activated *μ*-opioid receptors in the ENS inhibit muscle contractions by inhibiting the activity of the enzyme *AC* via their G_*i*_ protein and reducing the *cAMP* levels, leading to constipation.

Interestingly, an activation of *μ*-opioid receptors in the ENS leading to constipation is only partially accurate. Endogenous opioids, such as Endomorphin-2, do not induce the same constipating effects as pharmaceutical opioids, despite exhibiting high specificity for the *μ*-opioid receptor and showing similar potencies to Fentanyl in inhibiting *cAMP* accumulation in vivo [20,21]. The differences in how endogenous and pharmaceutical opioids affect the functionality of the ENS is due to their distinct biochemical properties. While pharmaceutical opioids are engineered for slow degradation, endogenous opioids, like Endomorphin-2, are naturally prone to rapid degradation [22,23,24]. This rapid degradation shortens their duration of action and prevents prolonged activation of *μ*-opioid receptors in the gut. In contrast, pharmaceutical opioids show prolonged degradation times, leading to longer lasting effects and also side effects, such as constipation.

Therefore, in the context of OIC, understanding and highlighting the differences between endogenous opioids and pharmaceutically relevant opioids may help elucidate the underlying causes and pave the way for providing new approaches for managing OIC.

OIC is a multifaceted process with numerous inter-related sub-pathways that have not been thoroughly examined by mathematical modeling. The current understanding of OIC primarily comes from clinical trials and in vitro studies, with clinical approaches predominantly focusing on treatment strategies rather than the underlying mechanisms [25,26,27]. In vivo studies suggest that opioids are dissimilar in pharmacodynamic profiles eliciting OIC with differential potencies, but the precise mechanisms are still unknown [28]. In contrast, mathematical models have been developed to explore the role of the neurotransmitter *5HT* within the enteric nervous system, including its metabolism and effects on gastrointestinal motility and constipation [12,29]. However, all these previously mentioned models typically examine a single pathway in isolation and do not account for the complex interactions between multiple competing pathways involved in OIC, such as those mediated by opioids and *5HT* together.

In this article, we present a mathematical model to investigate the effects of both clinical and endogenous opioids on constipation, with a focus on *AC* activity and *cAMP* accumulation. Furthermore, we explore these effects in the presence of an active serotonergic pathway, integrating the interplay between opioid and *5HT* signaling in the enteric nervous system. We reproduce previously published results and analyze both the *5HT* and opioid-induced pathways in relation to *AC* and *cAMP*, comparing our results to available literature. Additionally, we integrate these pathways to examine the impact of pharmaceutical opioids on an intact gut and contrast the effects with those of Endomorphin-2. Finally, we aim to offer new insights into addressing OIC by exploring characteristics of endogenous ligands, specifically Endomorphin-2. In particular, we seek to understand the effects of varying opioid degradation rates on *cAMP* recovery following acute opioid treatment.

## 2. Results

### 2.1. 5HT Has a Positive Cooperative Effect on cAMP Accumulation

**Figure 1:**
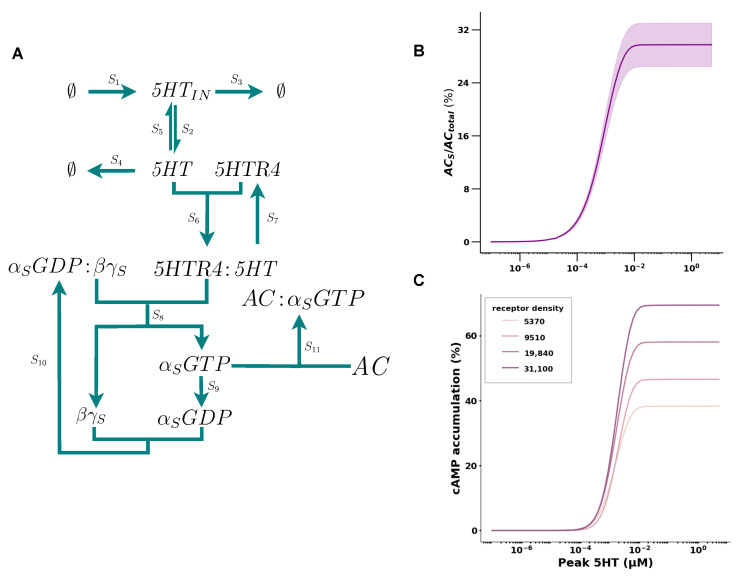
Mathematical model of the serotonin pathway. (**A**) Core reaction network of the serotonin pathway. Protein complexes labeled in text and reactions represented by arrows. To keep the reaction network more concise, $S_{12}$ has been omitted. See Table 3 for rates. (**B**) Dose response of *AC* activation for varying *5HT* concentrations. The window represents the range observed for varying *5HTR4* densities (5 × 10^−5^ μM and 5 × 10^−6^ μM). (**C**) Dose response of *cAMP* to varying *5HT* concentrations, where each curve represents different receptor densities. The curves are fits of the data given by [30].

We began by mathematically modeling the serotonin (*5HT*) pathway, starting with *5HT* release from the enterochromaffin (EC) cells to the cascade down to *AC* activation and the resulting accumulation of intracellular *cAMP* in enteric neurons. In the current literature, *AC* activity is not directly measured; rather, it is inferred through *cAMP* accumulation. As these two are treated to be synonymous, mathematical models of the intermediate reaction between *AC* and *cAMP* are scarce. To finalize our model for the *5HT* pathway, we proposed a Hill-type reaction from *AC* to *cAMP* and calibrated its parameters utilizing experimental evidence from the literature as follows:

We modeled the *5HT* pathway reactions S1–S12 as seen in Table 1 and Figure 1A. Since the *5HT* receptor (*5HTR4*) concentration is variable in vivo, we conducted simulations by sampling *5HTR4* concentrations in the interval between 5 × 10^−5^ μM and 5 × 10^−6^ μM. We found that the dose response of *AC* activation for varying concentrations of *5HT* showed a positive cooperative effect with an $EC_{50}$ of 0.33 ± 0.053 μM and a Hill coefficient (*n*) of 1.13 ± 0.017 (see Figure 1B). From the small standard error that we observed in the $EC_{50}$, we deduced that the *5HTR4* concentration did not effect the $EC_{50}$. In contrast, we observed that the range of $E_{\max}$ was modulated. In conclusion, the $EC_{50}$ of *AC* activity is not sensitive to *5HTR4* density; hence, we choose the receptor concentration of 5 × 10^−6^ (see Table 2) for the remainder of the simulations.

We then inferred the parameters of the reaction of *cAMP* accumulation by *AC* activation, which we modeled as a Hill function (see Section 4). For robustness, we inferred the reaction parameters from data of different receptor densities (see Figure 1C) and found that the different rates produced similar efficacy for *cAMP* accumulation (see Table A1 in Appendix A.1). Therefore, we choose the parameters $k_{1}=6.1\times 10^{-3}$, $k_{2}=141.3$, $EC_{50}=135$, and n=2.44, for the reaction for the simulation here in.

The addition of the *cAMP* production and degradation reactions C1 and C2 as given in Table 1 completes our mathematical model of the *5HT* pathway (S1–S12 in Table 1). In the next section, we will add reactions C1 and C2 to the opioid pathway and verify its consistency using data from the literature.

**Table 2:** List of the model species including their initial concentrations ${\vec{x}}_{0}$ and the corresponding source for these values.

Species (Reaction Network)	Species (COPASI)	Initial Concentration ${\vec{x}}_{0}$ in μM	Source
*5HT*	5HT	$10^{-7}$	Model Calibration Appendix A.2
*5HT_IN_*	5HT_IN	$1.8\times 10^{-4}$	Model Calibration Appendix A.2
*5HTR4*	5HTR4	$5\times 10^{-6}$	[31]
*AC*	*AC*	$2\times 10^{-34}$	Model Calibration Appendix A.2
5HTR4:5HT	5HTR4:5HT	0	
$AC\;:\;\alpha_{S}GTP$	AC:ALPHA_S_GTP	0	
$AC\;:\;\alpha_{I}GTP$	AC:ALPHA_I_GTP	0	
$\alpha_{S}GDP$	ALPHA_S_GDP	0	
$\alpha_{I}GDP$	ALPHA_I_GDP	0	
$\alpha_{S}GDP\;:\;\beta \gamma_{S}$	ALPHA_S_GDP_BG_S	0.05	Model Calibration Appendix A.2
$\alpha_{I}GDP\;:\;\beta \gamma_{I}$	ALPHA_I_GDP_BG_I	0.05	Model Calibration Appendix A.2
$\alpha_{S}GTP$	ALPHA_S_GTP	0	
$\alpha_{I}GTP$	ALPHA_I_GTP	0	
$\beta \gamma_{S}$	BG_S	0	
$\beta \gamma_{I}$	BG_I	0	
*MOR*	MOR	$5\times 10^{-6}$	[31]
MOR:OL	MOR:OL	0	
*OL*	OL	0	
$OL_{Blood}$	OL_Blood	0	

**Table 3:** List of reaction rate constants, along with a brief description of each, their corresponding values (including units), and the source.

Parameter	Description	Value	Source
$k_{\mathrm{synthesis}5HT_{IN}}$	Endogenous serotonin (in EC cell) synthesis rate constant	0.05 ms^−1^	Model calibration Appendix A.3
$k_{\mathrm{release}5HT_{IN}}$	Endogenous serotonin (in EC cell) release rate constant	0.001 ms^−1^	Calculated from [14]
$k_{\mathrm{degrade}5HT_{IN}}$	Endogenous serotonin (in EC cell) degradation rate constant	0.01 ms^−1^	Model calibration Appendix A.3
$k_{\mathrm{degrade}5HT}$	Cellular serotonin degradation rate constant	0.01 ms^−1^	Model calibration Appendix A.3
$k_{\mathrm{reuptake}5HT}$	Reuptake of intracellular serotonin into EC cell rate constant	$2.38\times 10^{-8}$ ms^−1^	Model calibration Appendix A.3
$k_{\mathrm{bind}5HT}$	Endogenous *5HT* binding to *5HTR4*	$4.46\times 10^{-3}$ μM^−1^·ms^−1^	Model calibration Appendix A.3
$k_{\mathrm{unbind}5HT}$	Endogenous 5HT unbinding from 5HTR4	$5\times 10^{-8}$ ms^−1^	Model calibration Appendix A.3
$k_{\mathrm{transfer}}$	Rate at which administered opioid reaches the desired tissue via the blood	$1.38\times 10^{-7}$ ms^−1^	Estimated from [32]
$k_{\deg OL}$	rate at which opioid in the desired tissue is metabolized	$1.02\times 10^{-2}$ ms^−1^	Estimated from [32]
$k_{\mathrm{elimination}}$	Rate at which opioid in the blood stream is eliminated	$1\times 10^{-8}$ ms^−1^	Estimated from [32]
$k_{\mathrm{bind}OL}$	Rate at which opioid binds to MOR	varying for opioids	Calculated from different sources Appendix A.3
$k_{\mathrm{unbind}OL}$	Rate at which opioid unbinds from MOR	varying for opioids	Calculated from different sources Appendix A.3
$k_{\mathrm{activation}}$	Rate at which $G_{\alpha }$ subunint is activated	0.2 μM^−1^·ms^−1^	Calculated from [33]
$k_{\mathrm{assembly}}$	Rate at which $G_{\alpha }$ and $G_{\beta \gamma }$ reform into G protein complex	0.033 μM^−1^·ms^−1^	Calculated from [33]
$k_{\mathrm{hydrolization}}$	Rate at which the GTP is hydrolyzed into a GDP	$6.7\times 10^{-5}$ ms^−1^	Calculated from [34]
$k_{AC\mathrm{association}}$	Rate at which *AC* and $G_{\alpha s}$ form a complex	$3.5\times 10^{-3}$ μM^−1^·ms^−1^	[35]
$k_{AC\mathrm{association}}$	Rate at which *AC* and $G_{\alpha i}$ form a complex	$3.5\times 10^{-3}$ μM^−1^·ms^−1^	[35]
$k_{AC\mathrm{dissociation}}$	rate at which $G_{\alpha s}$ dissociates from *AC*	$3.3\times 10^{-6}$ ms^−1^	Model calibration Appendix A.3
$k_{AC\mathrm{dissociation}}$	rate at which $G_{\alpha i}$ dissociates from *AC*	$3.3\times 10^{-6}$ ms^−1^	Model calibration Appendix A.3

### 2.2. The Activation of the Opioid Pathway Inhibits cAMP Accumulation

**Figure 2:**
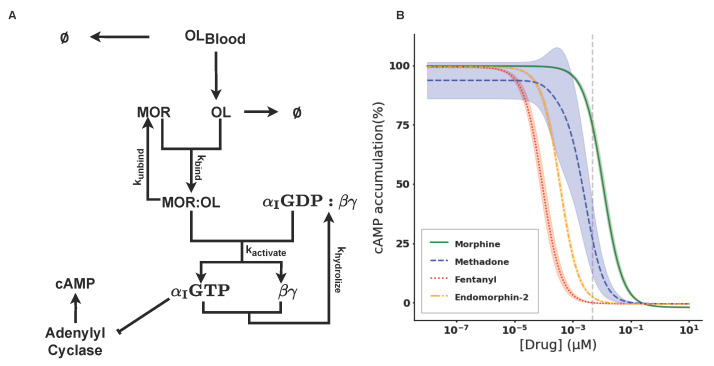
(**A**) Reaction network of the opioid pathway. Protein complexes labeled in text and reactions represented by arrows. To keep the reaction network more concise, $O_{10}$ has been omitted. (**B**) Dose response of *cAMP* to varying drug concentrations. The different curves represent the different drugs. Morphine is represented by a solid green line, Methadone by a blue dashed line, Fentanyl by a red dotted line, and Endomorphin-2 by an orange dash–dot line. $IC_{50}$ concentration of Morphine is highlighted by the gray dashed line.

**Figure 3:**
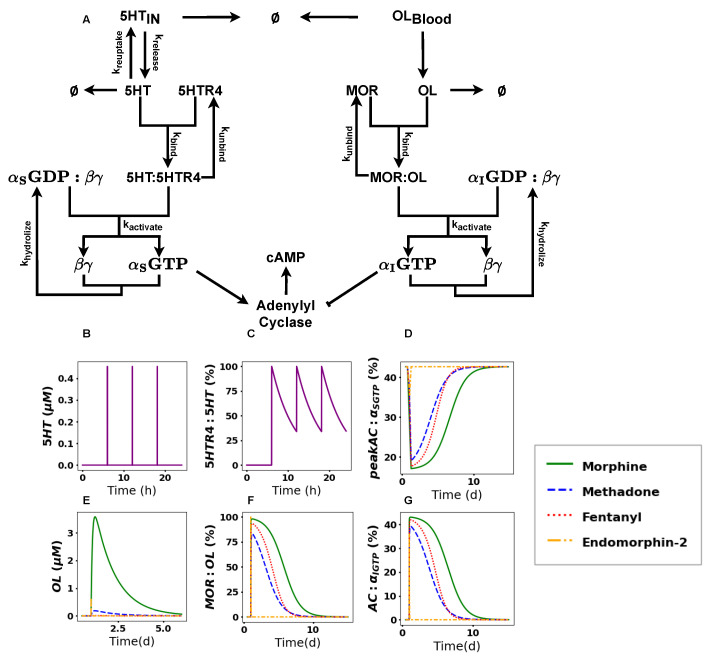
Model results for the competing reaction network after acute treatment with opioids. (**A**) Reaction network of the competing pathway. Protein complexes labeled in text and reactions represented by arrows. To keep the reaction network more concise $S_{12}$, $O_{10}$, and $C_{3}$ have been omitted. (**B**) Simulation results of *5HT* presented as concentrations (μM) over time (in hours). (**C**) Simulation results of *5HTR4*:*5HT* are presented as percentages over time (in hours). (**D**) Simulation results of peak $AC\;:\;\alpha_{S}GTP$ are presented as percentages over time (in days) for different drugs. Morphine is represented by a solid green line, Methadone by a blue dashed line, Fentanyl by a red dotted line and Endomorphin-2 by an orange dash–dot line. (**E**) Simulation results of OL are presented as concentrations (μM) over time (in days) for different drugs. Morphine is represented by a solid green line, Methadone by a blue dashed line, Fentanyl by a red dotted line, and Endomorphin-2 by an orange dash-dop are presented at line. (**F**) Simulation results for MOR:OL are presented as percentages over time (in days) for different drugs. Morphine is represented by a solid green line, Methadone by a blue dashed line, Fentanyl by a red dotted line, and Endomorphin-2 by an orange dash–dot line. (**G**) Simulation results for $AC\;:\;\alpha_{I}GTP$ are presented as percentages over time (in days) for different drugs. Morphine is represented by a solid green line, Methadone by a blue dashed line, Fentanyl by a red dotted line, and Endomorphin-2 by an orange dash–dot line.

The change in *cAMP* accumulation after the application of opioids has been studied experimentally; however, mathematical models that describe this pathway are very scarce. To build such a model, we adapted an opioid pathway model as seen in Figure 2A, by including two extra reactions. The included reactions are C1 and C2 (see Section 4 and Figure 3A). We then simulated the pathway for four different ligands and validated the results against the literature.

We investigated three drugs, Morphine, Methadone, and Fentanyl, and the endogenous opioid Endomorphin-2, all of which are full agonists with a high affinity for the *MOR*. To investigate the inhibition of *cAMP*, we started the simulation with all *AC* molecules switched on. This setup mimics the biological condition studied experimentally by [25], where all *AC* molecules in the cells were activated using forskolin.

All tested compounds showed a concentration-dependent inhibition of *cAMP* accumulation as seen in Figure 2B. Morphine, the standard opioid, showed the lowest potency with a $pIC_{50}$ of 7.95. Methadone, which is used as a substitution for patients with addiction, showed a $pIC_{50}$ of 8.7. Fentanyl, a highly potent opioid, showed the highest potency with a $pIC_{50}$ of 10.04 followed by the endogenous opioid, Endomorphin-2, with a $pIC_{50}$ of 9.43 (see Table 4).

We observed, with respect to *cAMP* accumulation, that Morphine was 10 times less potent than Methadone and 100 times less potent than Fentanyl. This is inline with the current literature. Surprisingly, the endogenous opioid Endomorphin-2 showed a similar order of magnitude in potency as Fentanyl, rather than Methadone or Morphine. Overall, the behavior of the *cAMP* inhibition curves observed from the simulations were in the order of magnitude observed in the literature (see Figure 2B and Table 4). With the serotonergic (S1–S12 in Table 1) and the opioid (O1–O10 in Table 1) model being defined, in the next section we combine them together and study the interaction of the two pathways.

### 2.3. cAMP Accumulation Drastically Decreases upon Acute Treatment with Opioids

It is known that opioids have an inhibitory effect on gut motility and secretion. Yet, the underlying mechanism in the presence of an active *5HT* pathway with respect to *cAMP* accumulation has not yet been mathematically modeled. To understand the effects of opioids on *cAMP* accumulation in the healthy gut, we combined all reactions from Table 1 as seen in Figure 3A. We simulated the effects of opioids on *cAMP* accumulation in the presence of an active *5HT* pathway for Morphine, Methadone, Fentanyl, and Endomorphin-2 with their equianalgesic initial doses of 10 mg (4.7 μM), 1mg (0.2 μM), 0.1 mg (0.01 μM), and 1425.51 μM, respectively. To capture the natural rapid decay of Endomorphin-2, we adjusted its degradation rate based on values from [22], decreasing it by a factor of $1\times 10^{-2}$. We added this extra factor to ensure a measurable decrease in *cAMP* accumulation levels. This adjustment seemed appropriate, as the rates from [22] were measured in vitro from different cellular compartments that may accelerate degradation. Hence, we selected one of their values as a benchmark. Since the pathways upstream from *AC* are independent from each other, the acute treatment of opioids did not affect the release of *5HT* and the binding kinetics of *5HT* to the *5HTR4* receptor (see Figure 3B,C). However, in the downstream reactions of the pathways, we observed a strong drug-dependent change in the peak concentrations of $AC\;:\;\alpha_{S}GTP$, which is the fraction of activated *AC* molecules (see Figure 3D).

We defined the threshold for recovery for *AC*. It was set to 80% of the maximal observation ($AC\;:\;\alpha_{S}GTP$) concentration. We defined the recovery time as the period required to reach 80% of the maximal observed activated *AC*($AC\;:\;\alpha_{S}GTP$) concentration. From this threshold, we observed that it took 7.8 ± 0.065 days for Morphine, 5.98 ± 0.30 days for Methadone, 5.25 ± 0.02 days for Fentanyl, and 6.2 h for Endomorphin-2 (see Figure 3D). With respect to the concentration of the drug present in the system, we observed that the drug depleted to its $IC_{50}$ concentration by 6.9 days for Morhphine, 5.14 days for Methadone, 4.58 days for Fentanyl, and 1 h for Endomorphin-2 (see Figure 3E).

Interestingly, we observed the number of bound receptors for the different compounds to be comparable. In particular, we observed 97.9% of the receptors being bound for Morphine, 93.3% for Fentanyl, 89.9% for Methadone, and 99.9% for Endomorphin-2 (see Figure 3F).

### 2.4. cAMP Shows Differences in Recovery Times

Without receptor internalization, the number of bound receptors gradually decreased for Morphine (12 days), Methadone (8 days), and Fentanyl (8 days); we observed a rapid decrease in bound receptors for Endomorphin-2 within a time span of 1 h (see Figure 3G).

The endogenous opioid Endomorphin-2 shows a high affinity towards the *MOR* and an equal potency as Fentanyl (see Table 4). However, its fast decay rate significantly limits the duration of its analgesic effect. With our mathematical model, we observed that the combination of high affinity and rapid drug decay led to a decrease in *cAMP* accumulation, lasting up to at most 6 h for Endomorphin-2. In contrast, the other opioids extended the decrease in *cAMP* accumulation for a significantly longer period, ranging between 4 and 7 days.

The short half life is very characteristic for endogenous ligands, as they show high specificity for their target receptors but are subject to rapid degradation. Building on this knowledge, we further investigated how variations in opioid degradation rates influence recovery times for *cAMP* accumulation under fixed drug concentrations and receptor affinities ($K_{d}$) in the next section.

### 2.5. Modulating Opioid Degradation Is an Alternative Approach to Improving the cAMP Recovery

**Figure 4:**
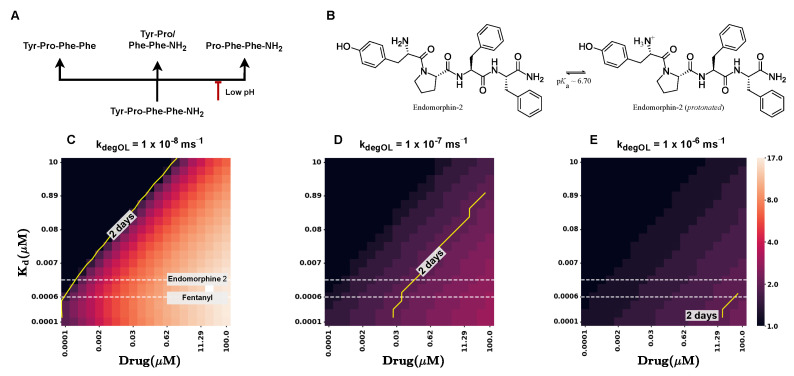
Effect of degradation on *cAMP* recovery times. (**A**) Degradation pathway of Endomorphin-2 by aminopeptidase, carboxypeptidase, or DPP IV. (**B**) Protonation of Endomorphin-2 in acidic pH. Recovery times of *cAMP* given $K_{d}$ and drug concentrations for $k_{degOL}=$$1\times 10^{-8}$ ms^−1^ (**C**), $k_{degOL}=$$1\times 10^{-7}$ ms^−1^ (**D**), and $k_{degOL}=$$1\times 10^{-6}$ ms^−1^ (**E**). White dashed lines depicting $K_{d}$ of Endomorphin-2 and Fentanyl. Yellow solid line marking the 2-day recovery time.

Endogenous opioids like Endomorphin-2 typically exhibit a short duration of action, mainly due to enzymatic degradation. In the case of Endomorphin-2, several enzymes contribute to its rapid breakdown, including aminopeptidase M, which cleaves at the amino terminus, carboxypeptidase Y which cleaves at the carboxyl end, and dipeptidyl peptidase IV (DPP IV), which removes N-terminal dipeptides (see Figure 4A) [22,41]. The enzymatic activity of these enzymes can vary based on several factors, one being the pH of the surrounding environment. We calculated the protonation of Endomorphin-2 in acidic environments at a pH of 6, as found in the gastrointestinal tract (see Section 4). We found that the aminoterminal nitrogen of Endomorphin-2 is protonated, which in turn increases the peptides’ stability (see Figure 4B). The protonationation of Endomorphin-2 may reduce the susceptibility to aminopeptidase cleavage (see Figure 4A; see red inhibition arrow), potentially leading to slower degradation in acidic environments such as those found in the gut.

To better understand the effects of degradation rates on *cAMP* levels, we investigated the impact of varying degradation rates $k_{\mathrm{degOL}}$. Specifically, we calculated *cAMP* recovery times by fixing the $k_{\mathrm{off}}$ rate, while varying the $k_{\mathrm{on}}$ rates and drug concentrations. These simulations were performed for three different degradation rates ($k_{\mathrm{degOL}}$) (see Figure 4C–E).

Figure 4C shows the recovery times for different $K_{d}$ values and drug doses for a degradation rate $k_{\mathrm{degOL}}$ of $1\times 10^{-8}$ ms^−1^, which corresponds to the degradation rate of conventional opioids such as Morphine, Methadone, and Fentanyl.

At this rate, recovery times are noticeably long. For a common drug dose of 0.01 μM for Fentanyl, we observed an average recovery time of 5 days.

As the degradation rate increased, we observed significant decreases in the recovery times. In particular, the recovery time for Fentanyl at a drug dose of 0.01 μM reduced to 3 days given $k_{degOL}$ being $1\times 10^{-7}$ ms^−1^ and less than 2 days for $k_{degOL}=$$1\times 10^{-6}$ ms^−1^ (see Figure 4D,E).

At this degradation rate, typical for conventional opioids, recovery times exhibited a strong dose-dependent increase (longer recovery times for higher doses). However, for faster degradation rates, this dose-dependent increase diminished, and recovery times remained relatively stable, even at higher doses (see Figure 4A–C).

In conclusion, our simulations indicate that accelerated degradation rates markedly decrease *cAMP* recovery times and diminish the dose-dependency of these recovery times. These data indicate that accelerated breakdown rates may facilitate larger medication dosing without a proportional increase in the severity of constipation. In the following section, we explore the broader implications of these results for opioid pharmacokinetics and potential therapeutic applications.

## 3. Discussion

In this work, we presented a robust mathematical model to explore the effects of opioids on opioid-induced constipation (OIC) with a focus on the change to adenylyl cyclase (*AC*) and *cAMP*.

First we considered the serotonergic and opioid pathways separately. We modeled the peristaltic nature of gut motility, to be in agreement with the existing literature. With respect to the opioid pathway, we were able to simulate the potencies of various opioids with respect to *cAMP* accumulation, using kinetic rates from previous studies. Our results indicate that Endomorphin-2 inhibits *cAMP* accumulation nearly as effective as Fentanyl when administered at equivalent doses [24].

**Figure A1:**
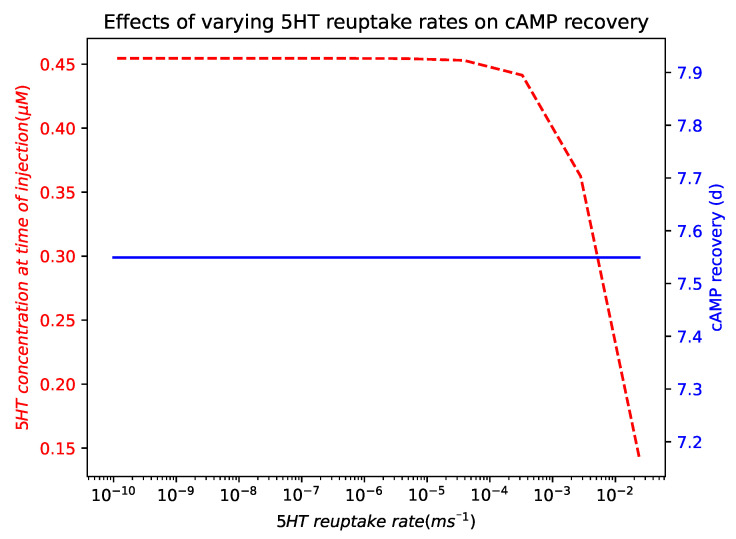
Effects of varying 5HT concentrations on cAMP recovery. The figure shows the effect of varying reuptake rates (ms^−1^) for cAMP recovery in the blue solid line and *5HT* concentrations at the time of drug injection (μM) in the red dashed line.

To explore the interplay between these two pathways, we integrated the serotonergic and the opioid pathway into a single reaction network, replicating the conditions of a healthy gut. Our analysis revealed that *5HT* levels and upstream signaling of adenylyl cyclase were not directly disrupted by acute opioid administration. In contrast, significant changes in adenylyl cyclase activity and *cAMP* levels were observed following acute opioid exposure. As opioids activate *μ*-opioid receptors according to their potency and concentration, they reduce gut motility, which can lead to an accumulation of gut contents and further disruption of healthy gut signaling. Since *5HT* is released in response to chemical or mechanical stimuli, the blockage of gut content would likely lead to an increase in *5HT* release, potentially resulting in excessive *5HT* levels within the gut environment. As a result, because the opioid pathway predominantly suppresses adenylyl cyclase activity via the G_*i*_ proteins, administering additional *5HT* does not resolve the issue of opioid-induced constipation and may, instead, increase the susceptibility to serotonin toxicity [42] (see Figure A1).

Therefore, to explore alternative approaches for addressing opioid-induced constipation, we examined the mechanisms of action of the endogenous opioid Endomorphin-2, which is not known to induce constipation in comparison to pharmaceutical opioids. Our analysis revealed no major differences in the affinities of Endomorphin-2 for the *μ*-opioid receptor compared to pharmaceutical opioids. In fact, the affinities suggested that Endomorphin-2 binds as strongly as the potent synthetic opioid Fentanyl. However, the main different characteristic between Endomorphin-2 and pharmaceutical opioids is the rapid degradation of Endomorphin-2. Endogenous opioids, including Endomorphin-2, are degraded significantly faster than commonly known opioids, and there are at least four known enzymes that degrade Endomorphin-2 [22,41].

Previous studies have attempted to enhance the stability of Endomorphin-2 to make it more suitable for medical use [24]. In our work, we demonstrated that Endomorphin-2 becomes protonated in an acidic environment, making it less susceptible to degradation by at least one enzyme which is aminopeptidase.

We suggest focusing on the possibilities of modulating the degradation of pharmaceutical opioids. Building on the work of [43], we suggest designing drugs that become active in specific environmental conditions, such as the acidic environments found in the gut. Such drugs could incorporate pH-sensitive enzymes that break down opioids selectively within the gut, minimizing systematic side effects. Additionally, we propose exploring complementary approaches, such as co-administration of an enzyme-containing drug that remains stable only in the gut compartment and is specific to degrading the corresponding pharmaceutical drug. It is important to note that the enzyme-containing drug should ideally be ingested after the opioid has been absorb into the blood, suggesting a shifted intake. Furthermore, it may be crucial to adjust the drug doses accordingly to ensure the analgesic effect. These proposed approaches could accelerate opioid degradation specifically within the gut, ideally mitigating opioid-induced constipation and maintaining their potency. Finally, we would like to emphasize that the cause of OIC is not solely mediated by peripherally activated *μ*-opioid receptors but also partially by activated *μ*-opioid receptors in the central nervous system. Nevertheless, targeting peripheral receptors appears to be a more feasible therapeutic strategy, with milder side effects.

Despite the valuable insights gained from our model, there are several limitations that should be acknowledged and which open opportunities for future developments. The accuracy of our simulations rely on kinetic rates obtained from the literature. These rates include the drug affinity ($K_{d}$) for the *μ*-opioid receptor. Reported values for the drug affinity $K_{d}$ often vary significantly across studies, even when similar experimental setups are used. This makes the parameter selection a complex process. To address this, establishing a standardized protocol to measure drug affinities across all known opioids would greatly enhance the precision of future iterations of the model.

Additionally, while our model focuses on a single dose, clinical practice typically involves extended administration that lasts over days or even weeks. However, as our model output currently focuses on *cAMP* accumulation levels, a single stimulus is sufficient for capturing the key dynamics we are studying and interested in. Introducing multiple doses would essentially result in longer recovery times and would not provide more valuable insights than what we have already gathered from studying the effects on *cAMP* for one single dose. Future iterations of our model should account for the effects of repeated dosing on physiological processes. In particular, it would be an important addition to incorporate receptor internalization. To achieve this, we propose the introduction of an additional species $MOR_{int}$, representing the internalized *μ* opioid receptor. Another key enhancement would be the quantitative mapping of *cAMP* accumulation to defecation, similar to the description in [12]. To achieve this, we propose introducing an additional compartment representing the downstream section of the colon, where defecation is regulated. The relationship between *cAMP* accumulation and defecation frequency could hereby be modeled using a Hill function providing a link between receptor signaling and physiological output.

Furthermore, the enteric nervous system is composed of multiple layers with various interacting neurons that span and connect these layers. Our current model, in particular, focuses only on motor neurons in the myentric plexus which are responsible for controlling the contractions of the longitudinal muscles. However, since it is known that neurons in other layers also express the *5HTR4* and the *μ*-opioid receptor [44,45], the model can be adapted and extended to represent neurons in different layers as well, broadening its applicability.

Lastly, it is important to keep in mind that the enteric nervous system (ENS) is highly complex and consists of other important pathways that are involved in it. For instance, it is known that both opioids and *5HT* influence other mechanisms beyond adenylyl cyclase and downstream *cAMP*. For example, the neurotransmitter *5HT* influences acetylcholine levels, while opioids are known to reduce calcium (Ca^2+^) levels [2,12]. These are just a few examples of the many interconnected processes steered by opioids and *5HT*. Nevertheless, our model takes an important step forward by integrating the serotonergic and opioid pathway into a single reaction network, rather than analyzing them separately.

To conclude, our model has proven valuable in unraveling the complexities and hidden mechanisms behind opioid-induced constipation, offering insights into understanding opioid-induced constipation. By shedding light on these underlying processes, our work paves the way for potential new strategies to address this challenging issue.

## 4. Methods and Materials

### 4.1. Reaction Network

The model simulates the direct effects of opioids and *5HT* on the enzyme *AC* via their respective GPCRs. The biochemical reaction network comprises 25 reactions, detailed in Table 1, and can be broadly divided into two pathways: the serotonergic pathway and the opioid pathway, both acting on the same downstream effector *AC*.

The endogenous neurotransmitter *5HT* is synthesized in the enterochromaffin (EC) cells of the gastrointestinal epithelium and is released in response to mechanical stimuli, such as the intake of a meal. Once released, *5HT* binds to *5HTR4* receptors located on motor neurons of the ENS, activating G protein type S. This activation induces the conversion of GDP to GTP and subsequently causes the dissociation of the G protein into its two subunits, with the $G_{\alpha S}$ subunit enhancing *AC* activity.

In contrast, in the opioid pathway, intravenously administered (or endogenously released) opioids travel through the bloodstream, with portions metabolized and portions distributed to the inflamed target tissue and non-target tissue such as the gut. In the gut, opioids bind to *μ*-opioid receptors on enteric neurons, activating G protein type I. This triggers the exchange of GDP to GTP and a dissociation of the G protein complex and a subsequent inhibition of *AC* activity by the $G_{\alpha I}$ subunit.

For both pathways, the mathematical model is given by the standard *reaction-rate equation* derived from the corresponding reaction network [46]. The reaction propensities are assumed to follow the law of mass action. Neglecting stochastic effects and assuming that the system is well mixed, we thus model the temporal evolution of species concentrations using a set of ordinary differential equations, with initial conditions specified at time t=0. The system of ordinary differential equations is given as follows:

(1) $$\frac{d\vec{x}\left(t\right)}{dt}=S\cdot \pi \left(\vec{x}\left(t\right),t\right),$$

with the initial conditions $\vec{x}\left(0\right)={\vec{x}}_{0}$ provided in Table 2, and the stoichiometric matrix S, the propensity functions $\pi (\vec{x}\left(t\right),t)$, and the species vector $\vec{x}\left(t\right)$ described in the Appendix A.4.

### 4.2. Software and Model Implementation

We utilized the biochemical simulator COPASI [47] to define the model and perform simulations, using the deterministic LSODA method [48]. Reaction rates were either directly obtained from published observations or estimated from them. A detailed list of the reaction rates and initial conditions is provided in Table 3. The simulations were automated in Python version 3.13.3 using the basico package [49]. Critical parameters, such as $EC_{50}$, which represents the concentration of the substance that induces a half-maximum response, and $IC_{50}$, which indicates the concentration necessary to inhibit a process by 50%, were inferred using the hillfit and HTS_doseresponse packages available at [50]. For parameter estimation, we applied the scipy.minimize function. The model file as well as the code required to reproduce the results of the analysis are available at: https://git.zib.de/sunkara/mathematical-model-for-oic (accessed on accesssed on 20 June 2025).

### 4.3. Mapping AC Activity to cAMP Production

*AC* activity is quantified by measuring *cAMP* accumulation over a defined interval. To enable meaningful comparison with existing datasets, it was necessary to convert *AC* activity into a *cAMP* accumulation readout. In our model, *AC* activity at time *t* is represented as the fraction of activated *AC* molecules relative to the total of *AC* molecules (including free, inhibited, and activated states):

(2) $$AC_{\mathrm{on}}\left(t\right)=\frac{AC\;:\;\alpha_{S}GTP\left(t\right)}{AC\;:\;\alpha_{S}GTP\left(t\right)+AC\;:\;\alpha_{I}GTP\left(t\right)+\mathrm{AC}\left(t\right)}.$$

Our model was extended to include two reactions: *cAMP* degradation, described by Michaelis Menten kinetics, and *cAMP* synthesis, modeled using a Hill function to account for cooperative binding, leading to the differential equation

(3) $$\frac{d}{dt}cAMP\left(t\right)=-k_{1}\cdot cAMP\left(t\right)+\frac{k_{2}{\left(\frac{AC_{\mathrm{on}}\left(t\right)}{EC_{50}}\right)}^{n}}{1+{\left(\frac{AC_{\mathrm{on}}\left(t\right)}{EC_{50}}\right)}^{n}},$$

for rate constants $k_{1},k_{2}>0$, and n>0 being the Hill coefficient. Here, cAMP(t) refers to the concentration of *cAMP* at time t≥0. In the context of molecule production, such as *cAMP* synthesis, the Hill function describes how the rate of *cAMP* synthesis depends on the fraction of activated *AC* molecules. The cooperative effects, encoded in the coefficient *n*, estimate how the quantity of activated *AC* molecules ($AC_{\mathrm{on}}$) influences the accumulation of *cAMP*. Higher *n* values indicate greater cooperativity.

We employed data from [30], where *cAMP* accumulation was measured for increasing *5HT* concentrations in cells expressing both wild-type and mutated *5HTR4*s, to derive the kinetic parameters for *cAMP* production ($k_{2}$) and degradation ($k_{1}$). We utilized [51] to map the inferred *AC* activity for a given *5HT* concentration in our model to the experimentally measured *cAMP* accumulation from [30]. By setting the left-hand side of Equation (3) to zero, we considered the system at steady state:

(4) $$0=-k_{1}\cdot cAMP+\frac{k_{2}{\left(\frac{AC_{\mathrm{on}}}{EC_{50}}\right)}^{n}}{1+{\left(\frac{AC_{\mathrm{on}}}{EC_{50}}\right)}^{n}}.$$

Calculating *cAMP* for the steady state of the subject, we get

(5) $$cAMP=\frac{k_{2}}{k_{1}}\;\frac{{\left(\frac{AC_{\mathrm{on}}}{EC_{50}}\right)}^{n}}{1+{\left(\frac{AC_{\mathrm{on}}}{EC_{50}}\right)}^{n}}.$$

Following Equation (5), we were able to infer the kinetic parameters $k_{1},k_{2},EC_{50}$ and the Hill coefficient *n* using scipy.minimize.

### 4.4. In Silico Protonation of Endomorphin-2

For estimating the most abundant protonation state of Endomorphin-2 under acidic conditions, we performed p*K_a_* predictions using QupKake [52]. This method combines graph neural network models with semiempirical quantum mechanical (QM) features for accurate micro-p*K_a_* predictions. QupKake predicted the basic micro-p*K_a_* of the N-terminal *a*-amino group at 6.696469. This indicated that at an acidic pH below 6.7, Endomorphin-2 is predominantly protonated at its N-terminus. We additionally performed protonation state predictions of Endomorphin-2 with Schrodinger’s Epik 7 Software [53]. Epik version 7 uses machine learning for predicting the p*K*_a_ values and protonation state distribution of molecules. Epik 7 predicted the aminoterminally to be the most populated state at an acidic pH of 6.0 ± 0.5.

## Figures and Tables

**Table 1:** List of reactions describing the reaction network on opioid-induced constipation. The network is divided into three main parts: the serotonergic pathway (denoted by *S*), the opioid pathway (denoted by *O*), and the competing pathway (denoted by *C*).

Compartment	*i*	$R_{i}$
Serotonin Pathway ($S_{i}$)	1	$\emptyset \overset{k_{\mathrm{synthesis}5{\mathrm{HT}}_{\mathrm{IN}}}}{\to }{5\mathrm{HT}}_{\mathrm{IN}}$*
	2	5HT *_IN_* $\overset{k_{\mathrm{release}5{\mathrm{HT}}_{\mathrm{IN}}}}{\to }5\mathrm{HT}$
	3	5HT *_IN_* $\overset{k_{\mathrm{degrade}5{\mathrm{HT}}_{\mathrm{IN}}}}{\to }\emptyset$
	4	$5\mathrm{HT}\overset{k_{\mathrm{degrade}5\mathrm{HT}}}{\to }\emptyset$
	5	$5\mathrm{HT}\overset{k_{\mathrm{reuptake}}}{\to }5\mathrm{HT}$ *_IN_*
	6	$5\mathrm{HT}+5\mathrm{HTR}4\overset{k_{\mathrm{bind}5\mathrm{HT}}}{\to }5\mathrm{HTR}4\;:\;5HT$
	7	$5\mathrm{HTR}4\;:\;5HT\overset{k_{\mathrm{unbind}5\mathrm{HT}}}{\to }5\mathrm{HTR}4$
	8	$5\mathrm{HTR}4\;:\;5HT+\alpha_{S}GDP\;:\;\beta \gamma_{S}\overset{k_{\mathrm{activation}}}{\to }5\mathrm{HTR}4\;:\;5HT+\alpha_{S}GTP+\beta \gamma_{S}$
	9	$\alpha_{S}GTP\overset{k_{\mathrm{hydrolization}}}{\to }\alpha_{S}GDP$
	10	$\alpha_{S}GDP+\beta \gamma_{S}\overset{k_{\mathrm{assembly}}}{\to }\alpha_{S}GDP\;:\;\beta \gamma_{S}$
	11	$\alpha_{S}GTP+\mathrm{AC}\overset{k_{\mathrm{ACactivation}}}{\to }AC\;:\;\alpha_{S}GTP$
	12	$AC\;:\;\alpha_{S}GTP\overset{k_{\mathrm{ACdissociation}}}{\to }\alpha_{S}GTP+\mathrm{AC}$
Opioid Pathway ($O_{i}$)	1	$\mathrm{OL}+\mathrm{MOR}\overset{k_{\mathrm{bind}\mathrm{OL}}}{\to }MOR\;:\;OL$
	2	$MOR\;:\;OL\overset{k_{\mathrm{unbind}OL}}{\to }\mathrm{MOR}$
	3	$OL_{Blood}\overset{k_{\mathrm{transfer}}}{\to }\mathrm{OL}$
	4	$OL_{Blood}\overset{k_{\mathrm{elimination}}}{\to }\emptyset$
	5	$\mathrm{OL}\overset{k_{\deg\mathrm{OL}}}{\to }\emptyset$
	6	$MOR\;:\;OL+\alpha_{I}GDP\;:\;\beta \gamma_{I}\overset{k_{\mathrm{activation}}}{\to }MOR\;:\;OL+\alpha_{I}GTP+\beta \gamma_{I}$
	7	$\alpha_{I}GTP\overset{k_{\mathrm{hydrolization}}}{\to }\alpha_{I}GDP$
	8	$\alpha_{I}GDP+\beta \gamma_{I}\overset{k_{\mathrm{assembly}}}{\to }\alpha_{I}GDP\;:\;\beta \gamma_{I}$
	9	$\alpha_{I}GTP+\mathrm{AC}\overset{k_{\mathrm{ACdeactivation}}}{\to }AC\;:\;\alpha_{I}GTP$
	10	$AC\;:\;\alpha_{I}GTP\overset{k_{\mathrm{ACIdissociation}}}{\to }\alpha_{I}GTP+\mathrm{AC}$
Competing Pathway ($C_{i}$)	1	$AC_{\mathrm{on}}\overset{k_{2}}{\to }cAMP$ see Equation (2)
	2	$cAMP\overset{k_{1}}{\to }\emptyset$
	3	$\alpha_{I}GTP+AC\;:\;\alpha_{S}GTP\overset{k_{3}}{\to }AC\;:\;\alpha_{I}GTP+\alpha_{S}GTP$

*5HT_IN_* denotes the concentration of serotonin in the enterochromaffin cells.

**Table 4:** Simulation results for inhibition of *cAMP* accumulation for various opioids. The affinities ($K_{d}$) were extracted from the literature and used to calculate the binding kinetics. The potencies are provided as $pIC_{50}$ values, along with a reference $pIC_{50}$.

Drug	$K_{d}$ (nM)	${\mathrm{pIC}}_{50}$ Simulations	Reference ${\mathrm{pIC}}_{50}$
Morphine	76.08 ± 9.19 [36]	7.95±0.018	6.7 [37]
Methadone	41.92 ± 28.31 [38]	8.7 ± 0.13	7.5 [37]
Fentanyl	0.57± 0.12 [39]	10.04 ± 0.03	8.9 [40]
Endomorphin-2	2.33 ± 0.19 [20]	9.43 ± 0.01	8.42 [23]

## Data Availability

The model file as well as the code required to reproduce the results of the analysis are available at: https://git.zib.de/sunkara/mathematical-model-for-oic (accesssed on 20 June 2025).

## Appendix A

### Appendix A.1. Reaction Parameters

**Table A1:** Fitted reaction parameters for cAMP production. $EC_{50}$ and $E_{\max}$ given for *cAMP* dose response to *5HT*.

Receptor Density	Fitted (k1, k2, ${\mathrm{EC}}_{50}$, n)	${\mathrm{EC}}_{50}$ (nM)	$E_{\max}$ (%)
5730	(0.1, 1.0, 2.1, 1.96)	1.37	38
9510	(0.0065, 14.14, 13.5, 2.44)	1.7	46
19,840	(0.018, 2.97, 6.2, 2.09)	1.49	58
31,100	(0.007, 50.25, 25.99, 2.24)	1.59	69

### Appendix A.2. Initial Conditions

The initial concentrations given in Table 2 were collated from the literature and also calibrated to the particular computational scenarios considered in this work. In particular

- We calibrated the initial concentration of *AC*, such that the *AC* activity is around 40%. It was shown by Das et al. that the baseline muscle contraction is in the human colon is around 40% [12].
- We calibrated the initial concentrations of *5HT* and *5HT_IN_* such that *5HT_IN_* is $10^{-2}$ smaller than *5HT* as estimated in [29].
- We calibrated the initial concentration of the receptor species *MOR* and *5HTR4* to that given in [31]. We calibrated the initial concentration of both $\alpha_{I}GDP$ and $\alpha_{S}GDP$ to ensure an approximate maximum *AC* activity of 40%.
- We calculated the equianalgesic doses for Morphine, Methadone, and Fentanyl from [6,54]. For Endomorphin-2 we used the slider option of COPASI to ensure that 100% of receptors are being bound.

### Appendix A.3. Parameter Settings

The reaction rates given in Table 3 were calculated from the literature and calibrated to the computational scenarios considered in this work. In particular

- Synthesis of *5HT_IN_* was calibrated using the COPASI sliders to ensure that the concentration ratio of *5HT_IN_* and *5HT* was greater than 1 as estimated in [29].
- We calibrated $k_{deg5HT}$ and $k_{deg5HT_{IN}}$ to ensure a ratio greater than 1 for *5HT_IN_* and *5HT*.
- We calibrated $k_{reuptake}$ to ensure a particular clearance time.
- We calibrated the binding and unbinding rate for *5HT* to ensure an $EC_{50}$ of around 0.05 μM, as implemented in Das et al.
- We calculated the binding and unbinding rates using $K_{d}$ values from available resources (see Table 4).
- We calibrated the reaction rates for $k_{activation}$, $k_{ACactivate}$, and $k_{ACdeactivate}$, such that the maximum amplitude of *AC* was capped at 40%, as suggested in Das et al.

### Appendix A.4. Stoichiometric Matrix

The species vector is given as follows:

$$\vec{x}\left(t\right):=\left(\begin{array}{c} 5{\mathrm{HT}}_{\mathrm{IN}}\left(t\right)\\ 5\mathrm{HT}(t)\\ 5\mathrm{HTR}4(t)\\ 5\mathrm{HTR}4\;:\;5HT(t)\\ \alpha_{S}GDP\;:\;\beta \gamma_{S}\left(t\right)\\ \beta \gamma_{S}\left(t\right)\\ \alpha_{S}GDP\left(t\right)\\ \alpha_{S}GTP\left(t\right)\\ OL_{Blood}\left(t\right)\\ \mathrm{OL}(t)\\ \mathrm{MOR}(t)\\ MOR\;:\;OL(t)\\ \alpha_{I}GDP\;:\;\beta \gamma_{I}\left(t\right)\\ \beta \gamma_{I}\left(t\right)\\ \alpha_{I}GDP\left(t\right)\\ \alpha_{I}GTP\left(t\right)\\ \mathrm{AC}(t)\\ AC\;:\;\alpha_{S}GTP\left(t\right)\\ AC\;:\;\alpha_{I}GTP\left(t\right)\\ \mathrm{cAMP}(t) \end{array}\right)$$

The propensity vector is given as follows:

$$\pi \left(\vec{x}\left(t\right),t\right):=\left(\begin{array}{c} k_{\mathrm{synthesis}5HT_{IN}}\\ k_{\mathrm{release}5HT_{IN}}\cdot 5{\mathrm{HT}}_{\mathrm{IN}}\left(t\right)\\ k_{\mathrm{degrade}5HT_{IN}}\cdot 5{\mathrm{HT}}_{\mathrm{IN}}\left(t\right)\\ k_{\mathrm{degrade}5HT}\cdot 5\mathrm{HT}\left(t\right)\\ k_{\mathrm{reuptake}}\cdot 5\mathrm{HT}\left(t\right)\\ k_{\mathrm{bind}5HT}\cdot 5\mathrm{HT}\left(t\right)\cdot 5\mathrm{HTR}4\left(t\right)\\ k_{\mathrm{unbind}5HT}\cdot 5\mathrm{HTR}4\left(t\right)\\ k_{\mathrm{activation}}\cdot \alpha_{S}GDP\;:\;\beta \gamma_{S}\left(t\right)\cdot 5\mathrm{HTR}4\;:\;5HT\left(t\right)\\ k_{\mathrm{hydrolization}}\cdot \alpha_{S}GTP\left(t\right)\\ k_{\mathrm{assembly}}\cdot \alpha_{S}GDP\left(t\right)\cdot \beta \gamma_{S}\left(t\right)\\ k_{AC\mathrm{association}}\cdot \alpha_{S}GTP\cdot \mathrm{AC}\left(t\right)\\ k_{AC\mathrm{dissociation}}\cdot AC\;:\;\alpha_{S}GTP\left(t\right)\\ k_{\mathrm{bind}OL}\cdot \mathrm{OL}\left(t\right)\cdot \mathrm{MOR}\left(t\right)\\ k_{\mathrm{unbind}OL}\cdot MOR\;:\;OL\left(t\right)\\ k_{\mathrm{transfer}}\cdot OL_{Blood}\left(t\right)\\ k_{\mathrm{elimination}}\cdot OL_{Blood}\left(t\right)\\ k_{\mathrm{degrage}OL}\cdot \mathrm{OL}\left(t\right)\\ k_{\mathrm{activation}}\cdot MOR\;:\;OL\left(t\right)\cdot \alpha_{I}GDP\;:\;\beta \gamma_{I}\left(t\right)\\ k_{\mathrm{hydrolization}}\cdot \alpha_{I}GTP\left(t\right)\\ k_{\mathrm{assembly}}\cdot \alpha_{I}GDP\left(t\right)\cdot \beta \gamma_{I}\left(t\right)\\ k_{AC\mathrm{association}}\cdot \alpha_{I}GTP\cdot \mathrm{AC}\left(t\right)\\ k_{AC\mathrm{dissociation}}\cdot AC\;:\;\alpha_{I}GTP\left(t\right)\\ k_{2}\cdot AC_{\mathrm{on}}\left(t\right)\;\mathrm{see}\;\mathrm{Equation}\;(2)\\ k_{1}\cdot \mathrm{cAMP}\left(t\right)\\ k_{3}\cdot \alpha_{I}GTP\left(t\right)\cdot AC\;:\;\alpha_{S}GTP\left(t\right) \end{array}\right)$$

The stoichiometric matrix is given as follows:

$$S=\left[\begin{array}{ccccccccccccccccccccccccc} 1 & -1 & -1 & 0 & 1 & 0 & 0 & 0 & 0 & 0 & 0 & 0 & 0 & 0 & 0 & 0 & 0 & 0 & 0 & 0 & 0 & 0 & 0 & 0 & 0\\ 0 & 1 & 0 & -1 & -1 & -1 & 0 & 0 & 0 & 0 & 0 & 0 & 0 & 0 & 0 & 0 & 0 & 0 & 0 & 0 & 0 & 0 & 0 & 0 & 0\\ 0 & 0 & 0 & 0 & 0 & -1 & 1 & 0 & 0 & 0 & 0 & 0 & 0 & 0 & 0 & 0 & 0 & 0 & 0 & 0 & 0 & 0 & 0 & 0 & 0\\ 0 & 0 & 0 & 0 & 0 & 1 & -1 & 0 & 0 & 0 & 0 & 0 & 0 & 0 & 0 & 0 & 0 & 0 & 0 & 0 & 0 & 0 & 0 & 0 & 0\\ 0 & 0 & 0 & 0 & 0 & 0 & 0 & -1 & 0 & 1 & 0 & 0 & 0 & 0 & 0 & 0 & 0 & 0 & 0 & 0 & 0 & 0 & 0 & 0 & 0\\ 0 & 0 & 0 & 0 & 0 & 0 & 0 & 1 & 0 & -1 & 0 & 0 & 0 & 0 & 0 & 0 & 0 & 0 & 0 & 0 & 0 & 0 & 0 & 0 & 0\\ 0 & 0 & 0 & 0 & 0 & 0 & 0 & 0 & 1 & -1 & 0 & 0 & 0 & 0 & 0 & 0 & 0 & 0 & 0 & 0 & 0 & 0 & 0 & 0 & 0\\ 0 & 0 & 0 & 0 & 0 & 0 & 0 & 1 & -1 & 0 & -1 & 1 & 0 & 0 & 0 & 0 & 0 & 0 & 0 & 0 & 0 & 0 & 0 & 0 & 1\\ 0 & 0 & 0 & 0 & 0 & 0 & 0 & 0 & 0 & 0 & 0 & 0 & 0 & 0 & -1 & -1 & 0 & 0 & 0 & 0 & 0 & 0 & 0 & 0 & 0\\ 0 & 0 & 0 & 0 & 0 & 0 & 0 & 0 & 0 & 0 & 0 & 0 & -1 & 0 & 1 & 0 & -1 & 0 & 0 & 0 & 0 & 0 & 0 & 0 & 0\\ 0 & 0 & 0 & 0 & 0 & 0 & 0 & 0 & 0 & 0 & 0 & 0 & -1 & 1 & 0 & 0 & 0 & 0 & 0 & 0 & 0 & 0 & 0 & 0 & 0\\ 0 & 0 & 0 & 0 & 0 & 0 & 0 & 0 & 0 & 0 & 0 & 0 & 1 & -1 & 0 & 0 & 0 & 0 & 0 & 0 & 0 & 0 & 0 & 0 & 0\\ 0 & 0 & 0 & 0 & 0 & 0 & 0 & 0 & 0 & 0 & 0 & 0 & 0 & 0 & 0 & 0 & 0 & -1 & 0 & 1 & 0 & 0 & 0 & 0 & 0\\ 0 & 0 & 0 & 0 & 0 & 0 & 0 & 0 & 0 & 0 & 0 & 0 & 0 & 0 & 0 & 0 & 0 & 1 & 0 & -1 & 0 & 0 & 0 & 0 & 0\\ 0 & 0 & 0 & 0 & 0 & 0 & 0 & 0 & 0 & 0 & 0 & 0 & 0 & 0 & 0 & 0 & 0 & 0 & 1 & -1 & 0 & 0 & 0 & 0 & 0\\ 0 & 0 & 0 & 0 & 0 & 0 & 0 & 0 & 0 & 0 & 0 & 0 & 0 & 0 & 0 & 0 & 0 & 1 & -1 & 0 & -1 & 1 & 0 & 0 & -1\\ 0 & 0 & 0 & 0 & 0 & 0 & 0 & 0 & 0 & 0 & -1 & 1 & 0 & 0 & 0 & 0 & 0 & 0 & 0 & 0 & -1 & 1 & 0 & 0 & 0\\ 0 & 0 & 0 & 0 & 0 & 0 & 0 & 0 & 0 & 0 & 1 & -1 & 0 & 0 & 0 & 0 & 0 & 0 & 0 & 0 & 0 & 0 & 0 & 0 & -1\\ 0 & 0 & 0 & 0 & 0 & 0 & 0 & 0 & 0 & 0 & 0 & 0 & 0 & 0 & 0 & 0 & 0 & 0 & 0 & 0 & 1 & -1 & 0 & 0 & 1\\ 0 & 0 & 0 & 0 & 0 & 0 & 0 & 0 & 0 & 0 & 0 & 0 & 0 & 0 & 0 & 0 & 0 & 0 & 0 & 0 & 0 & 0 & 1 & -1 & 0 \end{array}\right]$$

### Appendix A.5. Choice of Recovery Treshold

**Table A2:** Recovery times for $AC\;:\;\alpha_{S}GTP$ for different thresholds.

Treshold	Morphine (d)	Methadone (d)	Fentanyl (d)	Endomorphin-2 (h)
50%	6.162	4.446	4.177	6.191
57.5%	6.539	4.781	4.454	6.191
65%	6.891	5.117	4.705	6.191
72.5%	7.318	5.563	4.957	6.191
80%	7.796	5.982	5.259	6.191

### Appendix A.6. Effects of Varying 5HT Reuptake Rates on cAMP Recovery

Under the assumption that the *5HTR4* receptor number does not change, we studied the effects of increasing *5HT* concentrations on *cAMP* recovery, by modulating the *5HT* reuptake rate, the process by which *5HT* is reabsorbed into the intracellular depot. By decreasing the reuptake rate, we observed increasing *5HT* concentrations at the initial time of drug induction (see Figure A1 red dashed line), as was expected. However, surprisingly, an increase in the initial *5HT* concentrations had no effects on the *cAMP* recovery times (see Figure A1 blue solid line). In conclusion, increasing *5HT* concentrations has no effect on *AC* activation.
